# Symptomatic Pneumocephalus after Lumbar Disc Surgery: a Case Report

**DOI:** 10.3889/oamjms.2015.028

**Published:** 2015-02-16

**Authors:** Zahir Kizilay, Ali Yilmaz, Ozgur Ismailoglu

**Affiliations:** 1 *Adnan Menderes University, Medical Faculty, Neurosurgery, Aytepe Campus, Aydin 09100, Turkey*; 2 *Süleyman Demirel University, Neurosurgery, 32260 Isparta, Turkey*

**Keywords:** Lumbar disc surgery, Dura tear, Cerebrospinal fluid leakage, Vaccum suction device, Pneumocephalus

## Abstract

Symptomatic pneumocephalus is frequently seen after traumatic fracture of the skull base bone. However, it has rarely been reported after spinal surgery and its mechanism has not been fully explained. In this paper, we present a 30 year old male patient who had lumbar discectomy due to a symptomatic midline lumbar disc herniation. He had developed symptomatic pneumocephalus after the lumbar disc surgery associated with application of a vacuum suction device. We present and discuss our patient in the light of the literatures.

## Introduction

Many complications due to spinal surgery have been reported in the literature. While most of these complications are diagnosed intraoperatively, only a small proportion of them are diagnosed in the postoperative period. Cerebrospinal fluid (CSF) leakage is among the most common complications encountered in spinal surgery practice and its prevalence is 0.3-5.9% [1]. Pneumocephalus and pneumoarrchachis is a problem which is rarely encountered and its cause has not been fully revealed.

## Case Presentation

**Figure 1:**
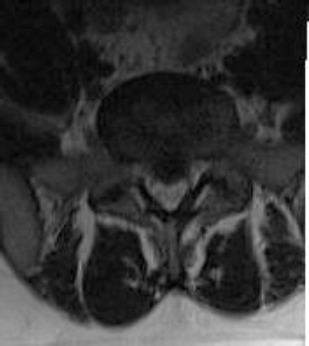
*L5-S1 midline lumbar disc herniation in axial section MRI*.

A 30-year-old male patient was admitted with pain in the lower back and left leg. There was no motor deficit on neurological examination. Laseque was 40 degrees in the left and 60 degrees in the right. Lumbar Magnetic Resonance Imaging (MRI) revealed an extruded disc fragment in the midline of at L5-S1 level (Fig. 1).

**Figure 2:**
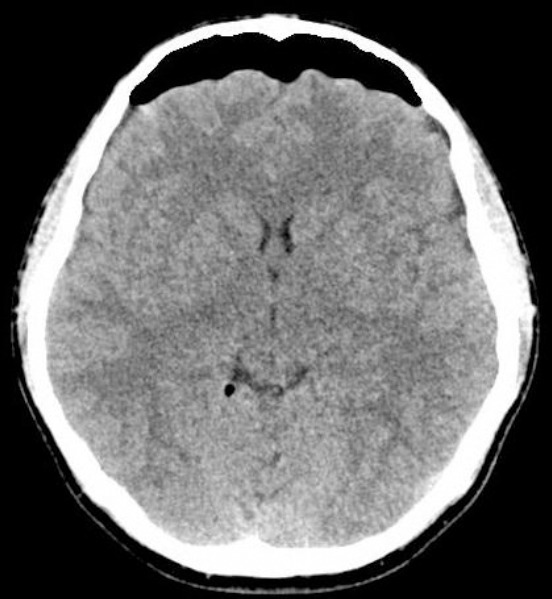
*Symptomatic pneumocephalus in axial cranial CT obtained in postperative period*.

**Figure 3:**
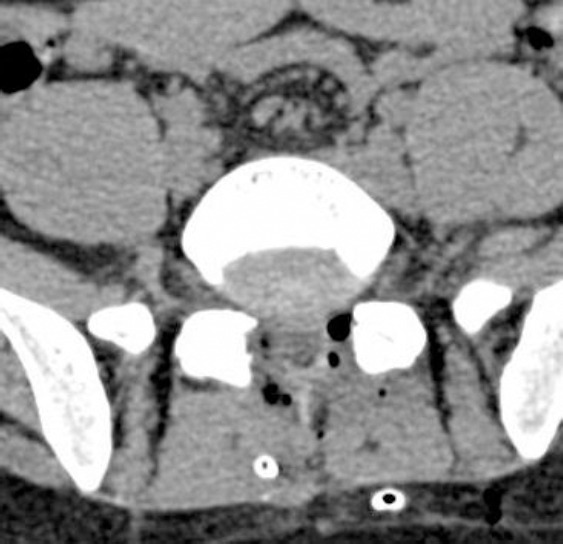
*The appearance of the air in laminectomy field and the under-fascia part of the lumbar vacuum suction device on postoperative lumbar CT*.

Surgical treatment was considered because the low back and leg pain was irresponsive to medical treatment and physical therapy and because of the very large midline disc. Bilateral hemilaminectomy was performed due to the midline disc. Dura injury was observed on the ventral surface when left discectomy was being performed. A certain amount of CSF leakage was observed just after the dura tear and it was seen to stop spontaneously after a while. A sponge was placed and no other treatment was performed (as Tissel). An unsettled vacuum suction device was placed under the fascia on the left considering that a future CSF fistula might develop as bilateral hemilaminectomy was performed, and abundant haemorrhage developed during discectomy. Fascia and subcutaneous tissue were tightly sutured. A total of 200 cc CSF-blood mixture was seen to flow from the vacuum suction device of the patient who had bed rest for two days. The patient was mobilized on postoperative day two and, experienced headache and nausea, vomiting, but he did not have a stiff neck or fever, so infection was not considered after evaluation by an infectious diseases specialist, and bed rest was recommended again. Cranial and lumbar computed tomography (CT) was obtained on postoperative day four. When his complaints continued, cranial CT revealed intracranial pneumocephaly (Fig. 2) and lumbar CT revealed air under profound fascia and at the wound site (Fig. 3).

The vacuum suction device was removed on postoperative day five as no flow or wound problem was observed and the skin was sutured. The patient’s complaints completely regressed on postoperative day six and he was discharged on postoperative day eight.

## Discussion

The development of diagnostic tools like CT and MRI have enabled spinal surgeons to evaluate preoperative pathologies, to plan the operation and to recognize secondary complications developing due to intraoperative and postoperative complications. Specific and non-specific complications are now being reported in the literature due to increasing spinal approaches. For example, an incidence of dura injury of 0.3-5.9% in spinal surgery practice may show that surgeons frequently encounter CSF fistula [1]. Although many complications secondary to CSF fistula have been reported, spinal surgery-related symptomatic pneumocephalus has rarely been reported.

Pneumocephaly is defined as the presence of air under the calvarial bones, or in the epidural, subdural, subarachnoid or intraventricular space [2, 3]. It is commonly seen in brain stem fractures or complicated open skull fractures together with dura and arachnoid injury secondary to head trauma, and it may also be seen due to tumour or infection or following lumbar puncture procedures performed for surgical or diagnostic purposes [2, 4]. In the literature, only seven cases with symptomatic pneumocephalus due to spinal surgery have been reported, but its frequency was not reported [1-7].

Four hypotheses have been proposed for the pathophysiology of pneumocephalus. One of these is the reverse bottle mechanism. The continuing CSF leakage causes negative intracranial pressure and thereby air replaces CSF until the pressure in the air and in the intracranial space become equal. The second mechanism is the ball valve hypothesis. This hypothesis proposes that pneumocephalus is caused by the air pressure in the paranasal sinuses exceeding intracranial pressure due to coughing, sneezing or swallowing. The third hypothesis proposes that it could occur during anaesthesia due to diffusion of nitric oxide (N_2_O). In this hypothesis, gas-producing bacteria are reported to cause pneumocephalus [2].

Although many hypotheses have been proposed to explaining symptomatic pneumocephalus when seven symptomatic pneumocephalus cases developing after posterior spinal surgery were analysed, dural injury was detected in five cases but not detected in two, and a vacuum suction device was used in five cases (Table 1).

**Table 1:** Previous published cases.

Authors	Age	Gender	Dural Tear	Drain	Treatment
Özturk et al [5]	23	F	+	+	Conservative
Turgut and Akyuz [4]	47	M	+	+	Conservative
Yun et al [2]	59	M	+	-	Conservative
Ayberk et al [3]	55	F	-	+	Conservative
Nowak et al [7]	12	F	+	+	Surgical
Dhamija et al [6]	63	F	+	-	Conservative
Karavelioglu et al [1]	56	M	-	+	Conservative
Present case	30	M	+	+	Conservative

Ayberk et al. reported that even though there no intraoperatively detected dura defect, indirectly increasing intracranial pressure secondary to increased intra-abdominal pressure due to prone position in obese patients opens an occult defect around the cribriform endplate and may lead to tension pneumocephalus [3]. Karavelioglu et al. also reported a case in which the dura tear could not be shown intraoperatively. Researchers reported that a part of a calcified sequestered disc fragment leaning downwards could lead to a dura tear in the axillary perineurium of the root, and this tear site could work as a one direction valve system after the disc had been removed and consequently lead to pneumocephalus [1]. Karavelioğlu et al. reported that a vacuum suction device could be a predisposing factor for pneumocephalus development, as did Turgut et al. [1, 4]. In addition, dura tearing was observed in six out of seven cases reported, but no information was given abouth bringing the patients into a modified Trandelenburg position (prone and head down position) in the intraoperative period [1-7]. It has been reported in the literature that the modified Trandelenburg position reduces the risk of pneumucephaly [2].

In our case, the presence of CSF leakage in the intraoperative period and its spontaneous termination after a while suggests the reverse bottle mechanism. The presence of air at the operation site and the vacuum drain on the postoperative lumbar CT suggests that the vacuum suction device could be a predisposing factor for symptomatic pneumocephalus. Why does symptomatic pneumocephalus not develop in many patients who have dura tear? Possible causes of this include using a vacuum suction device in dura and arachnoid tear cases with or without CSF leakage in the intraoperative period, long lasting muscle retraction (there may be a potential space under the fascia), repairing dura tear over a long time, a long operative time, extreme reduction of intracranial pressure secondary to dura tear and, arachnoid injury in patients who have borderline moderate intracranial pressure.

Clinical findings may be silent or non-specific and also may include headache, nausea, vomiting, lethargy, photophobia and meningeal irritation [2-7]. X-ray graphy, cranial CT and MRI may be used as diagnostic tools. The capability of detecting 0.5 cc of air enables the use of CT as a highly specific and sensitive diagnostic tool [1, 2, 6]. We used CT to make a diagnosis of pneumocephalus and detected pneumocephalus mainly in the frontal region. The patient was treated conservatively with bed rest, iv hydration and iv antibiotic therapy as in six out of seven cases in the literature.

In conclusion, the modified Trendelenburg position should be applied whenever dural tearing and cerebrospinal fluid escape occurs. Dura tear should be closed as quickly as possible in order to prevent symptomatic pneumocephalus that could develop due to dura and arachnoid injury during lumbar disc surgery. Dura tear should be sutured as quickly as possible. Closing the tear with fascia or muscle, surgicel, sponge and use of the synthetic adhesives like albumin, collagen and gluteraldehyde are of great importance in reducing risk of intracranial pneumocephalus. Another important point is to keep the vacuum suction device unsettled until wound recovery in patients who are placed on a vacuum suction device and the risk for intracranial pneumocephalus should be kept in mind when the patient is being followed up.
